# Organic Afterglow Materials for Tumor Diagnosis and Therapy

**DOI:** 10.3390/bios15080484

**Published:** 2025-07-25

**Authors:** Xiayi Chen, Bin Li, Baoli Yin, Dong Xu, Youjuan Wang

**Affiliations:** 1 The Key Laboratory of Zhejiang Province for Basic and Clinical Application of Functional Nucleic Acids, Hangzhou Institute of Medicine (HIM), Chinese Academy of Sciences, Zhejiang Cancer Hospital, Hangzhou 310022, China; chenxiayi1114@163.com (X.C.); 18561806346@163.com (B.L.); 2 State Key Laboratory of Chemo/Biosensing and Chemometrics, College of Chemistry and Chemical Engineering, Hunan University, Changsha 410082, China; yinbl@hnu.edu.cn

**Keywords:** afterglow luminescence imaging, organic afterglow nanoparticles, tumor diagnosis and therapy

## Abstract

Organic afterglow nanoparticles (OANs) represent a unique class of optical materials capable of sustaining luminescence after excitation cessation. Owing to their exceptional design flexibility, tunable optical properties, and favorable biosafety profiles, OAN-based afterglow imaging has emerged as an advanced modality in tumor diagnosis and therapy. These nanostructures demonstrate significant potential in guiding precision surgical interventions and real-time monitoring of tumor treatment, including photodynamic therapy, photothermal therapy, and immunotherapy. This review systematically analyzes and discusses the luminescence mechanisms of OANs under various excitation sources, with particular emphasis on recent developments in tumor detection and treatment. Additionally, we also discuss the current challenges and future perspectives of using these nanoparticles in this field.

## 1. Introduction

As a prominent bioimaging modality, optical imaging enables high-resolution visualization of both anatomical structures and physiological processes. Relative to traditional imaging techniques (e.g., magnetic resonance imaging (MRI) and computed tomography (CT) scans), optical imaging exhibits higher sensitivity and specificity, lower invasiveness, and improved adaptability for both structural and functional in vivo analysis. This distinctive advantage significantly enhances the fidelity and productivity of experimental studies, pathological assessment, and treatment regimens [1]. However, traditional optical imaging methods such as fluorescence imaging rely on real-time excitation sources and are susceptible to interference from autofluorescence in biological tissues [2]. Although bioluminescence and chemiluminescence imaging eliminate the requirement for real-time excitation, they are still limited by enzyme-substrate kinetics [3]. To overcome these challenges, afterglow imaging, a unique luminescence phenomenon, enables the emission of luminescence persistently even after the excitation source is removed. This process is characterized by a half-life ranging from seconds to hours, and allows for recurrent excitation [4]. In contrast to phosphorescence (triplet-state emission) and delayed fluorescence (thermally activated reverse intersystem crossing), both involving short-lived exciton transitions, afterglow luminescence is sustained post-excitation through oxygen-mediated reactions that slowly decompose high-energy intermediates. Most importantly, because afterglow luminescence does not require real-time excitation, it presents several unique benefits, including significantly reduced autofluorescence background, enhanced imaging contrast with high signal-to-background ratios (SBR), and extended luminescence persistence for improved detection [5]. These features have made afterglow imaging a growing research focus in bioimaging and therapy fields (Table 1).

Given these remarkable advantages of afterglow imaging, the development of high-performance afterglow luminescent materials has become crucial for realizing its full biomedical potential. Afterglow luminescent materials are fundamentally categorized into inorganic and organic systems [6,7]. The field of inorganic afterglow materials originated with SrAl_2_O_4_ in the 19th century [8]. Through crystal engineering and doping strategies, researchers have developed transition metal (TM)- and lanthanide (Ln)-based nanomaterials with improved afterglow properties [9]. However, their biomedical applications are constrained by inherent drawbacks, including heavy metal ion leakage risks, non-biodegradability, structural rigidity, and energy-intensive synthesis, which pose significant biosafety concerns and may lead to inflammatory responses or organ accumulation, ultimately limiting clinical translation. Organic afterglow nanoparticles (OANs) have emerged as promising biomedical materials due to their tunable structures and excellent biocompatibility. A significant advancement occurred in 2015 when Pu developed the first MEHPPV (poly[2-methoxy-5-(2-ethylhexyloxy)-1,4-phenyl-enevinylene])-based afterglow system from poly (p-phenylenevinylene)s (PPVs) derivatives [10]. Subsequent studies expanded the scope to thiophene-functionalized polymers (PFODBT) [11] and small molecules like Schaap’s dioxetanes [12], enabling bioimaging applications. Recent progress includes porphyrin-based compounds (Ppa, Ce4) for diagnostics and therapy [12,13], as well as trianthracene derivatives—now leading candidates for deep-tissue imaging owing to their exceptional photon yield.

**Table 1:** Performance comparison of organic afterglow materials and traditional imaging agents.

	Examples	Real-Time Excitation Light	λ_em_ [nm]	Biocompatibility	Biological Applications	Ref.
**Organic Afterglow Materials**	MEHPPV	Not Required	595	Good	Lymph nodes, tumors and drug-induced hepatotoxicity imaging	[7,10]
	PFODBT		690		Real-time monitoring of photodynamic therapy, visualization of immune response and tumor imaging	[14]
	mMB		710		Early detection of inflammation, peritoneal metastatic tumors imaging and intraoperative navigation	[15]
	Ce4		680		Peritoneal metastatic tumors imaging and intraoperative navigation	[13]
	TAD		625–650		Colorectal cancer, glioblastoma and other tumors imaging, Granzyme B imaging and vascular lesion visualization	[16]
**Inorganic** **Afterglow Materials**	SrAl_2_O_4_	Not Required	520	Poor	Thermoluminescence and photoconductivity research	[8]
	ZnSn_2_O_4_:Cr,Eu(ZSO)		800		Breast cancer imaging and tumor targeting capacity	[9,17]
	NaGd (Y) F_4_:Ln@NaGd (Y) F_4_(Ln = Er/Tm/Ho/Nd)		1525, 1475, 1180, 1064		Vascular and tumor imaging and real-time ureter tracking	[9,18]
	mSiO_2_@Zn_0.6_Ca_0.4_Ga_2_O_4_:Cr^3+^,Yb^3+^		650–800		Tumor imaging and dynamic monitoring	[9,19]
**NIR dyes**	ICG	Required	780–830	Good	Hepatic function assessment, vascular imaging and tumor surgical navigation	[20,21]
	BODIPY		NIR-I(700–900), NIR-II(1000–1700)		Photodynamic/photothermal therapy, deep tissue imaging and biosensors	
	IR-780		780–820		Tumor photothermal therapy, fluorescence-guided surgery and drug delivery monitoring	
	SOD9-TPP		600–800		Antioxidant therapy, mitochondrial protection and oxidative stress imaging	
**Quantum dots**	PbS	Required	800–1800	Poor	Tumor imaging, biosensors and photodynamic therapy monitoring	[22,23]
	CdSe		500–1000		Fluorescence labeling, biological imaging and nucleic acid testing	
	InAs		1000–3000		Vascular and tumor imaging, biocompatible devices	
	Ag2Te		1000–1300		Tumor angiogenesis detection, drug delivery tracking, photothermal therapy monitoring and biosensors	

Given these advantages, OANs are poised to play an indispensable role in advancing biomedical research [24]. In tumor diagnostics, OANs enable precise lesion localization with high sensitivity and outstanding signal-to-background ratio, allowing clear delineation of tumor infiltration margins and providing reliable clinical diagnostic information [16,25,26,27]. As for tumor therapy, OANs function not only as therapeutic agents and nanocarriers for chemotherapeutic drugs but also enable real-time monitoring of drug release kinetics through their afterglow signals, thereby facilitating dynamic treatment optimization [13,28,29]. Currently, researchers have systematically enhanced the afterglow performance of these materials via molecular engineering strategies, successfully developed multifunctional theranostic platforms that have achieved notable progress in in vivo imaging, intraoperative navigation, and personalized therapy. These advancements offer innovative solutions for precision oncology.

**Scheme 1:**
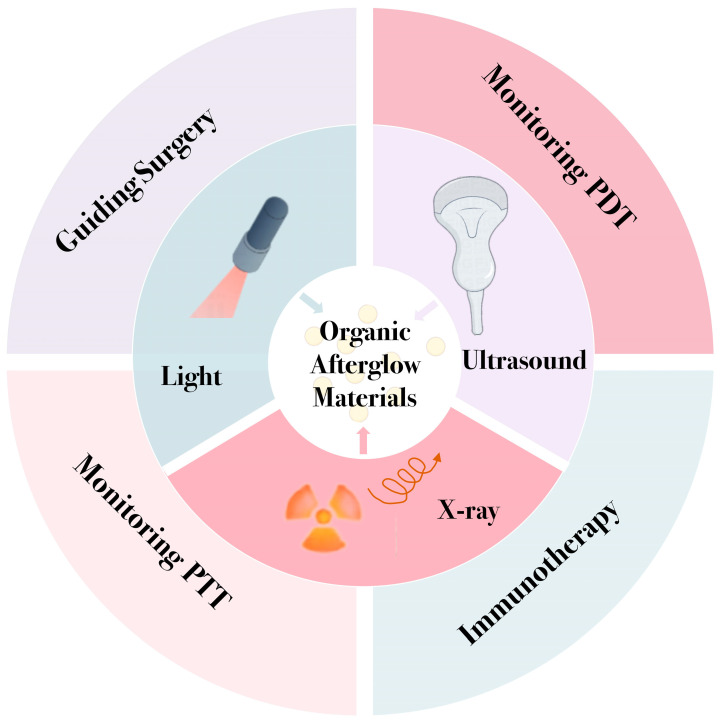
Scheme illustrating excitation sources and biomedical applications of organic afterglow nanoparticles.

This review provides an overview of the luminescence mechanisms of OANs under different excitation conditions, including light, ultrasound, and X-ray, with a focus on their latest research progress and applications in tumor therapy (Scheme 1). Additionally, we will discuss the challenges these nanoparticles face in biomedical applications and explore future directions. The goal is to offer new perspectives for researchers, thereby advancing the application and development of afterglow luminescence technology in biomedicine.

## 2. Light-Induced Afterglow Imaging

Light serves as the primary excitation source for afterglow luminescent materials, and its mechanisms can be broadly categorized into physical and chemical mechanisms. In the physical mechanism, afterglow luminescent materials, upon the absorption of light energy, facilitate electronic transitions across various energy levels through the generation and stabilization of excitons, leading to afterglow luminescence. The observed phenomena arise from: (i) radiative relaxation from singlet excited states to ground states, coupled with (ii) dynamic equilibrium between triplet and singlet states established through competitive intersystem crossing (ISC) and reverse intersystem crossing (RISC) processes [30,31]. In the chemical mechanism, light energy is converted and sequestered within unstable cyclic peroxides as chemical energy, which is incrementally released as photons upon the decomposition of these peroxides [10,32,33,34]. Thus far, these dual mechanisms have been extensively utilized in the domain of afterglow luminescence.

In recent years, the domain of OANs has seen remarkable progress. The following discussion will meticulously elucidate the performance characteristics and photoluminescence mechanisms of three distinct categories of organic nanoparticles.

### 2.1. Semiconducting Polymers (SPs)

Poly (p-phenylenevinylene)s (PPVs), a member of the semiconducting polymers (SPs) family, is an organic nanoparticle distinguished by its unique optical properties. The core structure of PPVs, which confers these special characteristics, is based on the p-phenylenevinylene motif. As early as 2015, a derivative of PPVs was first reported as a material exhibiting afterglow properties [10].

**Figure 1:**
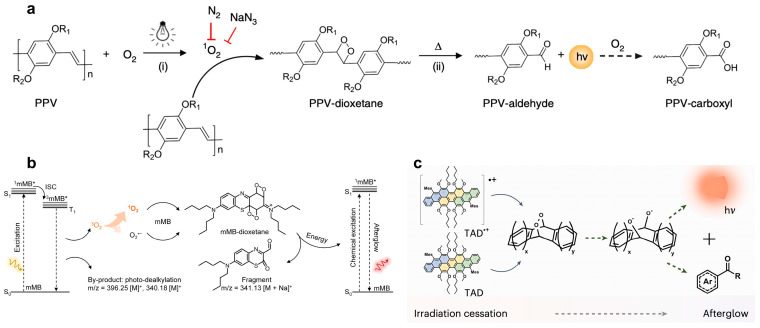
Light-induced luminescence mechanisms of different organic nanoparticles. (**a**) Luminescent mechanism of PPV-based semiconducting polymers (R1/R2: alkyl chains). Reprinted with permission from Ref. [7]. Copyright 2017 Springer Nature America, Inc. (**b**) Chemiluminescence pathway of mMB system (m/z data presented as mean ± SD, *n* = 3), mMB*, excited mMB. Reprinted with permission from Ref. [15]. Copyright 2024 Wiley-VCH GmbH. (**c**) Molecular design, synthesis, and principle of TAD-NPs afterglow system. Reprinted with permission from Ref. [16]. Copyright 2024 Springer Nature.

Miao et al. [7] have identified that PPVs, when adorned with electron-donating alkoxy substituents like BOPPV, MDMOPPV, and MEHPPV, manifest observable afterglow luminescence. The underlying mechanism involves the photogeneration of singlet oxygen (^1^O_2_) upon irradiation, which engages in a π^2^–π^2^ cycloaddition with the vinylene bond (C=C) of the PPVs, yielding a highly reactive PPV-dioxetane intermediate. This intermediate, being inherently unstable, undergoes spontaneous degradation into a PPV-aldehyde, in the process emitting photons. Photocatalytic oxidation of PPV-aldehyde ultimately generates PPV-carboxyl as the terminal reaction product (Figure 1a).

**Figure 2:**
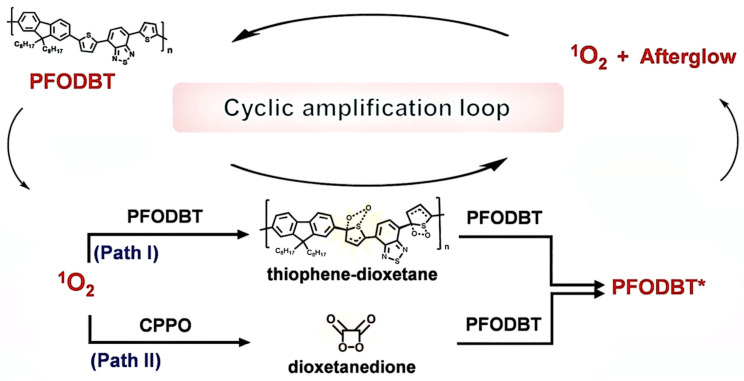
Afterglow luminescence mechanism studies. Investigation of the delayed emission mechanism in PFODBT@CPPO nanoparticles. PFODBT*, excited PFODBT. Reprinted with permission from Ref. [14]. Copyright 2021 Wiley-VCH GmbH.

Concurrently, our team conducted studies on organic semiconductors incorporating thiophene groups. We developed PFODBT@CPPO nanoparticles via nanoprecipitation, exhibiting 80–100 nm diameter and buffer stability. Optimization of CPPO doping and Pluronic-F127 surfactant enhanced NIR afterglow intensity 8-fold over PFODBT alone. PFODBT functioned as: (1) photosensitizer generating ^1^O_2_ via ISC; (2) substrate forming thiophene-dioxetane intermediate; and (3) chemically initiated electron exchange luminescence (CIEEL)-based energy relay for NIR emission. CPPO amplified signals through ^1^O_2_-triggered dioxetanedione formation, creating a dual cyclic loop where ^1^O_2_ activated both components, intermediate cleavage excited more PFODBT, and PFODBT regenerated ^1^O_2_ via ISC. This synergy achieved 8-min afterglow duration, 7.5-mm tissue penetration, and 16× higher SBR than fluorescence imaging (Figure 2) [14].

### 2.2. Heterocyclic Compounds

Among various OANs, heterocyclic structures—particularly methylene blue and porphyrin derivatives—have garnered significant research interest due to their exceptional photoactive characteristics.

Zhu et al. [15] pioneered the investigation of methylene blue’s (mMB) persistent luminescence behavior. Their findings revealed that mMB serves as a multifunctional component in the afterglow system, acting simultaneously as a luminescence trigger, reaction medium, and energy transfer mediator, thereby amplifying near-infrared chemiluminescence. The proposed photophysical process can be described as follows: When irradiated, mMB generates abundant ^1^O_2_ along with trace amounts of superoxide radicals (O_2_^•−^). These reactive oxygen species facilitate the oxidative cleavage of the dual vinylene linkages in the aromatic side chain, producing an mMB-dioxetane transition state. This transient high-energy species rapidly undergoes decomposition, accompanied by light emission (Figure 1b).

In addition to mMB, porphyrins (such as Ce4, Ce6, and Ppa), with their highly conjugated systems and unique photoelectric properties, have found widespread application in clinical practice. Taking Ce4 as an example [13], Ce4 absorbs energy and transfers it to oxygen, producing ^1^O_2_. The generated ^1^O_2_ subsequently oxidizes the vinylene bond (C=C) through a π^2^-π^2^ cycloaddition, producing a Ce4-dioxetane intermediate. This intermediate then decomposes to generate the observed afterglow luminescence.

### 2.3. Trianthracene Derivatives (TADs)

To address the limited triplet energy that constrains ^1^O_2_ production and subsequent afterglow emission, we developed an innovative trianthracene derivative (TAD) through rational molecular engineering [16]. Our team strategically incorporated alkoxy substituents at both ortho- and bay-positions of the anthracene core to augment electron donation capability. The TAD’s unique afterglow behavior arises from the cooperative interaction between two distinct photodynamic processes: The first involves electron transfer reactions that generate O_2_^•−^ and TAD radical cations (TAD^•+^), while the second proceeds through energy transfer to produce ^1^O_2_ alongside ground-state TAD molecules. Importantly, the reactive species created via the electron transfer pathway synergistically enhance the efficiency of the energy transfer mechanism, forming a self-reinforcing cycle that dramatically improves intermediate production and afterglow performance (Figure 1c). Experimental evaluations demonstrated the TAD’s remarkable afterglow intensity, surpassing MEHPPV by approximately 500-fold, while also exhibiting superior tissue penetration characteristics. These exceptional properties establish TADs as highly promising candidates for next-generation afterglow applications.

## 3. Ultrasound-Induced Afterglow Imaging

The implementation of light-induced luminescence for deep-tissue imaging faces significant limitations due to photon scattering and reabsorption effects in biological media. Ultrasound, as a noninvasive mechanical energy source with excellent deep tissue penetration capability, can effectively transfer its energy to luminescent nanoparticles through a well-characterized mechanoluminescence mechanism, thereby generating a persistent afterglow emission. This method fundamentally overcomes the depth limitations associated with traditional optical excitation methods while maintaining excellent spatiotemporal resolution [35,36]. Therefore, ultrasound-induced afterglow luminescence shows great potential in promoting precision medicine in the diagnostic monitoring of visceral organs.

### 3.1. Sonoafterglow Nanoparticles (SNAPs)

The ultrasound-induced afterglow luminescence system is composed of three critical elements: a sonosensitizer, a luminescent substrate, and a stabilizer. Upon ultrasonic activation, the sonosensitizer produces ^1^O_2_ through an energy transfer process. This reactive oxygen species (ROS) oxidizes the luminescent substrate, generating a high-energy intermediate that undergoes controlled decomposition, thereby releasing stored chemical energy as delayed luminescence. The stabilizer ensures nanoparticle structural integrity and promotes dispersion and stability in physiological environments. Through systematic screening of sonosensitizers and substrates, compositional optimization, and incorporation of biocompatible stabilizing agents, Xu et al. [37] established a robust platform, named sonoafterglow nanoparticles (SNAPs), for efficient afterglow imaging.

**Figure 3:**
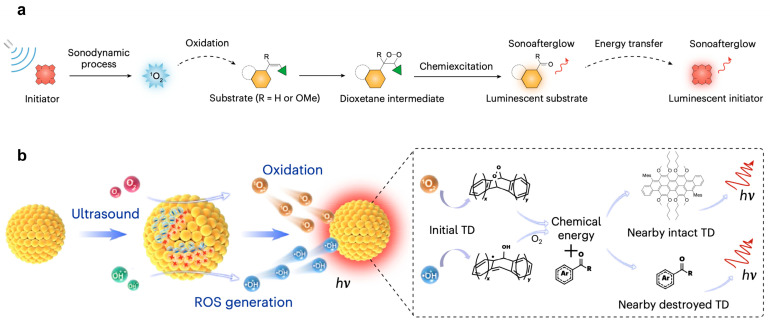
Mechanism of ultrasound activated luminescence. (**a**) Sonochemical activation pathway: Ultrasound-triggered ^1^O_2_ generation initiates dioxetane formation from substrates, followed by gradual decomposition yielding persistent emission. Energy recycling occurs through sensitizer re-excitation with spectral red-shift. Reprinted with permission from Ref. [37]. Copyright 2022 Springer Nature. (**b**) Piezoluminescent process in TD nanoparticles: Mechanically-induced photon emission (hν) via piezoelectric transduction. Reprinted with permission from Ref. [38]. Copyright 2024 Nature Photonics.

The fundamental process underlying ultrasound-induced afterglow luminescence in SNAPs is clearly delineated (Figure 3a). Initially, the sonosensitizer absorbs acoustic energy, generating ^1^O_2_ via physicochemical transduction. This ^1^O_2_ then catalyzes the conversion of the substrate into an energy-rich intermediate that emits photons upon decomposition. Notably, the emitted photons can be reabsorbed by the sonosensitizer and re-emitted at longer wavelengths through energy transfer, resulting in signal amplification. This mechanism enhances both luminescence intensity and persistence. Optimization of the sonosensitizer-to-substrate ratio and ultrasound parameters enabled imaging at tissue depths up to 4 cm, surpassing conventional photoafterglow limitations. The system’s high sensitivity and deep-tissue penetration establish its potential for high-contrast, real-time imaging in biomedical diagnostics.

### 3.2. Molecules Luminescence Through Two-Step Intraparticle Energy Conversion

Differing from above mentioned ultrasound-induced luminescence system, our team have pioneered organic molecules leveraging a piezoelectric effect to achieve ultrasound-induced afterglow luminescence [38]. Employing a trianthracene derivative (TD) as both the luminescent molecule and piezoelectric component, the approach utilizes a nano-precipitation method to encapsulate TD into a hydrophobic core (TD NPs), forming spherical nanoparticles with an average diameter of 30–40 nm.

When subjected to ultrasound irradiation, TD NPs exhibit piezoelectric polarization that drives catalytic generation of reactive oxygen species (^1^O_2_ and •OH) through piezocatalytic processes. These generated ROS then react with TD NPs to form metastable dioxetane-based high-energy intermediates. The subsequent decomposition of these intermediates results in efficient energy release through photon emission (Figure 3b). The developed system demonstrates a remarkable 2389.6-fold enhancement in ultrasound-triggered luminescence intensity compared to conventional water-based sonoluminescence. This significant increase in luminescence intensity directly contributes to its superior imaging performance, achieving a spatial resolution of 1.46 mm and a tissue penetration depth of 2.2 cm. Moreover, leveraging this enhanced luminescence capability, the platform is engineered to enable dual-mode imaging, seamlessly integrating both immediate and delayed ultrasound-induced luminescence imaging modalities, thereby providing more comprehensive and detailed information for various applications.

## 4. X-Ray-Induced Afterglow Imaging

X-ray, a type of electromagnetic radiation, consist of high-energy photons with extremely short wavelengths, allowing them to penetrate atomic structures. This unique property enables X-ray to effectively deliver energy to deep tissue areas, enhancing energy storage for afterglow luminescence [39]. Based on the unique tunability and structural flexibility of OANs, X-ray-induced afterglow luminescence characteristics offer an innovative technological pathway for achieving precise radiotherapy.

**Figure 4:**
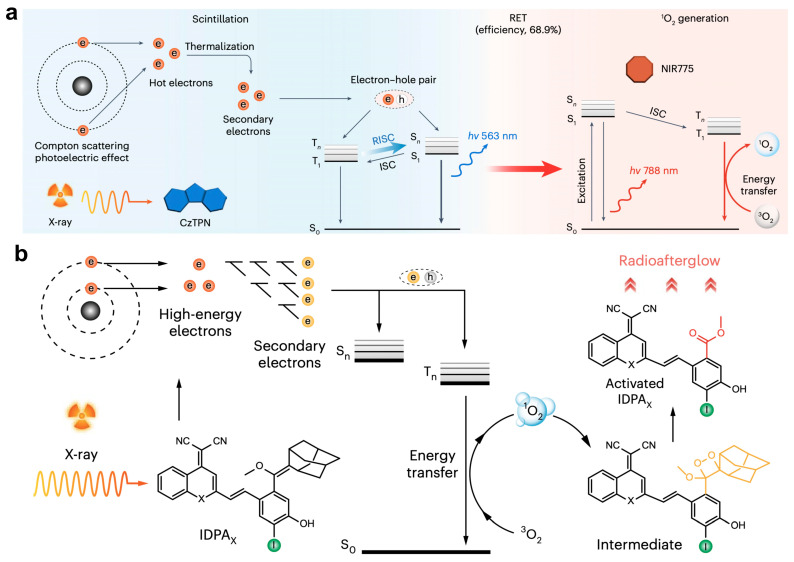
Mechanism of X-ray-activated luminescence. (**a**) X-ray energy conversion pathway in RANP: The radioabsorber CzTPN transforms X-ray energy into visible luminescence through photoelectric absorption and Compton scattering. This scintillation energy is subsequently relayed to NIR775, enabling near-infrared emission and ^1^O_2_ production via ISC/RISC processes. Reprinted with permission from Ref. [40]. Copyright 2024 Springer Nature. (**b**) Radio-afterglow generation process: Initial X-ray absorption by IDPA_X_ creates high-energy photoelectrons that undergo sequential energy loss, forming thermalized charge carriers. I/S-rich environments promote triplet exciton formation, whose decay energizes molecular oxygen to yield ^1^O_2_. This reactive species initiates dioxetane formation and subsequent photon release through cyclic peroxide breakdown. Reprinted with permission from Ref. [4]. Copyright 2023 Springer Nature.

Xu et al. [40] have proposed a cascade X-ray energy conversion system for X-ray induced afterglow luminescence, utilizing a radioabsorber (CzTPN) that attenuates X-ray through photoelectric and Compton effects. The X-ray energy undergoes conversion to radioluminescent emissions, which subsequently activate the radiosensitizer (NIR775) to produce ^1^O_2_. The ^1^O_2_ can effectively diffuse to the radio-afterglow substrates, where it oxidizes them into an active dioxetane intermediate. After the cessation of X-ray exposure, these intermediates gradually decompose, emitting radio-afterglow. The emitted photons can also be transferred back to NIR775, resulting in a red-shifted radio-afterglow from the radiosensitizer (Figure 4a).

Moreover, the team also reported a type of OANs [4] that are composed of an organic luminophore (IDPAsu), a tumor-targeting moiety, and an enzyme-cleavable peptide. These OANs demonstrate a radio-afterglow signal enhancement exceeding 25-fold relative to traditional inorganic phosphors, while simultaneously generating a significantly higher yield of ^1^O_2_. The mechanism begins with X-rays interacting with the atoms in IDPAx through photoelectric and Compton effects, leading to the production of high-energy hot electrons. These electrons then interact with nearby atoms, generating low-energy secondary electrons and electron-hole pairs. Under the influence of iodine and sulfur, a substantial number of electrons are transferred into triplet excited states. During the excitonic transition to ground state, they transfer their energy to surrounding oxygen molecules, producing ^1^O_2_. The in situ-generated ^1^O_2_ subsequently undergoes a cycloaddition reaction with IDPAx, forming a dioxetane intermediate. Finally, this intermediate decomposes into the corresponding induced IDPAx, releasing photons and resulting in afterglow emission (Figure 4b).

## 5. Organic Afterglow Nanoparticles for Cancer Therapy

The distinctive benefits of OANs have led to their broad adoption across various biomedical research applications in recent years. Numerous studies have reported the development of innovative nanoplatforms based on these nanoparticles, which not only exhibit exceptional optical properties but also facilitate precise tumor-targeted imaging and real-time monitoring of therapeutic responses. These capabilities hold significant promise for advancing early cancer diagnosis and enabling more personalized treatment strategies.

### 5.1. Afterglow Imaging Guiding Surgery

Accurate intraoperative identification of a tumor’s location and its invasive margins is crucial for improving treatment outcomes and prognosis. By interacting with specific biomarkers in tumor cells, OANs generate bright islands at the tumor site, thereby enabling real-time imaging to provide precise guidance during tumor surgery.

**Figure 5:**
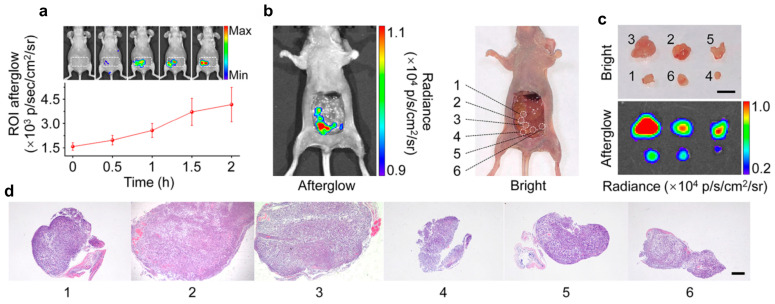
Afterglow imaging of tiny metastatic tumors in vivo together with the histological analysis of the tumors extracted. (**a**) Temporal afterglow imaging series following intravenous administration of NPs-Ce4 in murine models, with quantified luminescence intensities (ROI analysis, n = 3). (**b**) Dual-mode visualization combining afterglow and bright-field imaging post NPs-Ce4 injection (200 μg/mL, 200 μL dose). (**c**) Comparative ex vivo imaging of resected tumors showing bright-field (**upper**) and afterglow (**lower**) modalities (5 mm scale). (**d**) Histopathological correlation via H&E-stained tumor sections (400 μm scale) corresponding to (**c**). Reprinted with permission from Ref. [13]. Copyright 2022 American Chemical Society.

Chen et al. [13] reported the development of an organic afterglow nanoparticle (NPs-Ce4) for in vivo tumor imaging. Following intravenous administration, the light-induced NPs-Ce6 enabled continuous monitoring of distinct afterglow signals in the abdominal region, with a notably high SBR (Figure 5a). Additionally, histopathological analysis confirmed that, guided by the afterglow signal, surgical resection of tumor tissues was performed with enhanced precision, particularly in the detection of small tumors, where the technique demonstrated significant efficacy in identifying tumor lesions measuring just 3 mm^3^ in volume (Figure 5b–d).

A high signal-to-background ratio combined with high sensitivity proves crucial for cancer surgery guidance by allowing visualization of minute and residual tumor lesions [41]. To achieve enhanced afterglow luminescence signals, Duan et al. [29] developed organic nanoparticles (Ppa-FFGYSA) that form β-sheet structures via supramolecular self-assembly, remarkably amplifying both photoacoustic and afterglow luminescence signals. These nanoparticles leverage dual targeting capability combining passive the enhanced permeability and retention (EPR) accumulation with EphA2 receptor-specific binding to generate robust photoacoustic signals at tumor sites. Moreover, afterglow luminescence imaging, free from tissue autofluorescence interference, enables clear detection of submillimeter tumor nodules in the complex peritoneal environment, demonstrating excellent spatiotemporal controllability and imaging performance.

### 5.2. Afterglow Imaging Monitoring Photodynamic Therapy

Photodynamic therapy (PDT) represents a spatially targeted anticancer strategy where the interaction between a light-induced photosensitizer and tissue oxygen generates cytotoxic reactive oxygen species, enabling precise tumor eradication with minimal invasiveness [42]. Compared to traditional fluorescence imaging, afterglow luminescence imaging can provide real-time information with a high SBR, thereby offering robust support for precise therapy. Therefore, the development of an imaging system that integrates afterglow luminescence imaging with PDT has become a research hotspot in this field [43,44].

**Figure 6:**
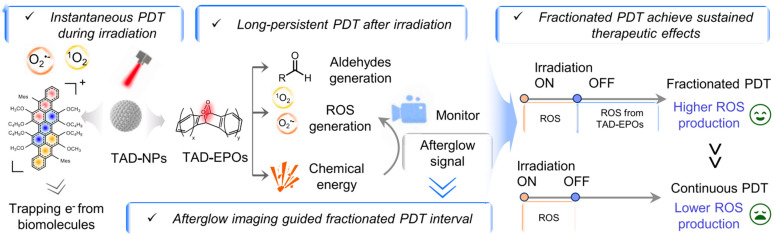
Illustration showing the working principle of TAD-NP-mediated divided-dose photodynamic therapy in tumor cells. Reprinted with permission from Ref. [28]. Copyright 2024 American Chemical Society.

In addressing the challenges of hypoxic conditions within solid tumors that limit the efficacy of PDT by impeding the generation of ROS, we proposed an innovative fractionated PDT protocol [28]. Our team developed an innovative TAD-based photosensitizer engineered to maintain ROS production persistently following light withdrawal, thereby inducing an afterglow photodynamic effect. The protocol involved administering light irradiation through intermittent illumination phases with recovery periods for oxygen replenishment, while employing afterglow imaging to track continuous ROS generation. Furthermore, we optimized the therapeutic outcomes of PDT by meticulously controlling the intervals between irradiation sessions, the dosage of illumination, along with the irradiation cycle duration (Figure 6). This strategy extended the duration of therapeutic effects and reduced damage to normal tissues, demonstrating significant promise for clinical application.

### 5.3. Afterglow Imaging Monitoring Photothermal Therapy

Photothermal therapy (PTT) employs photoconversion materials that effectively transform optical energy into thermal energy, thereby annihilating tumor cells with abbreviated treatment durations and minimal side effects. To minimize thermal damage to surrounding healthy tissues, a plethora of OANs with high photothermal conversion efficiencies have been reported. These OANs harness afterglow imaging to monitor the photothermal temperature of tumors, thereby guiding PTT. However, the majority of these nanoparticles predominantly absorb light in the NIR, which is characterized by limited tissue penetration capabilities [45].

**Figure 7:**
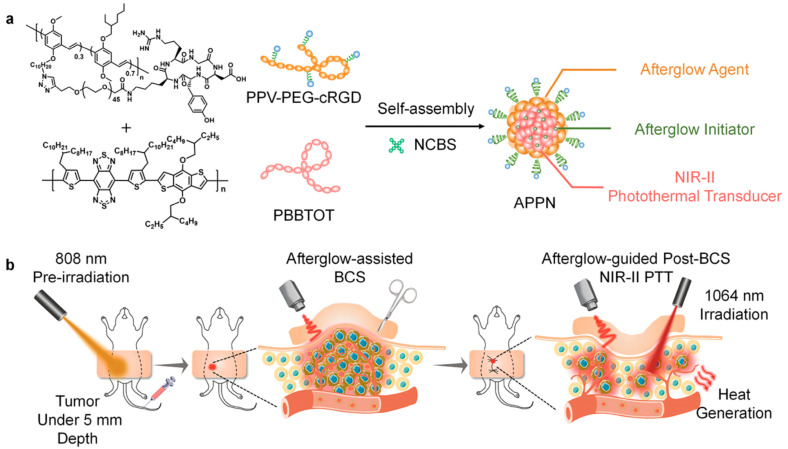
(**a**) Structural formulas of PPV-PEG-cRGD and PBBTOT compounds and preparation scheme for APPN assembly. (**b**) Procedural overview showing intraoperative afterglow imaging during BCS and subsequent NIR-II photothermal treatment guided by persistent luminescence. Reprinted with permission from Ref. [46]. Copyright 2023 American Chemical Society.

Qu et al. [46] engineered a dual-functional polymeric nanosystem (APPN) incorporating three key components: a tumor-targeted afterglow probe (PPV-PEG-cRGD), an afterglow activator (NCBS), and a NIR-II photothermal converter (PBBTOT) (Figure 7a). The APPN nanoparticles leverage 808 nm laser pre-irradiation to activate NCBS, which produces ^1^O_2_ that interacts with PPV’s vinylene groups to yield dioxetane species. These high-energy compounds gradually emit photons during decomposition, producing near-infrared afterglow through fluorescence resonance energy transfer-mediated energy transfer for accurate tumor visualization. Simultaneously, APPN achieves efficient photothermal conversion (80.82%) under 1064 nm laser irradiation, facilitating deep-tissue PTT with excellent thermal stability. This multifunctional platform enables precise afterglow imaging and NIR-II PTT, specifically eliminating remaining malignant cells to prevent tumor regrowth (Figure 7b). APPN’s distinctive characteristics allow for high-contrast imaging and effective PTT, presenting a viable strategy for adjuvant treatment following breast-conserving surgery.

### 5.4. Afterglow Imaging Immunotherapy

Immunotherapy, a strategy that harnesses the body’s immune system to combat tumor cells, has become increasingly effective against recurrent and refractory tumors [47]. However, due to individual patient variability and the toxicity associated with drug overdose, the efficacy of immunotherapy has not yet reached an optimal level [48]. By developing specific afterglow imaging techniques targeting immune response-related biomarkers [37], it enables the visual monitoring of immune cell activity throughout the immunotherapy process.

**Figure 8:**
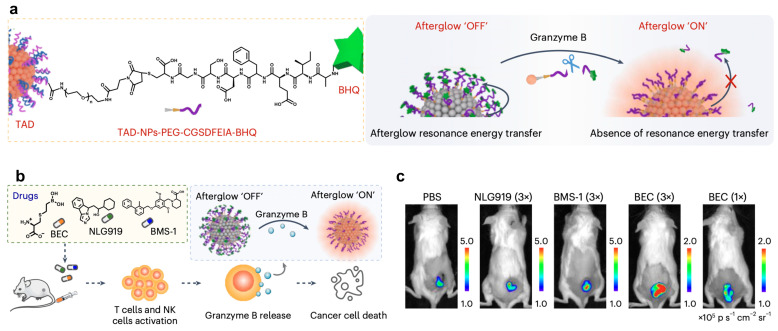
Afterglow imaging of immune responses after chemotherapy in vivo. (**a**) Working mechanism of TAD-BHQ nanocomplex for granzyme B-responsive afterglow visualization. (**b**,**c**) Afterglow monitoring in subcutaneous CT-26 tumors after treatment regimens: PBS control, triple-dose NLG919/BMS-1/BEC, single-dose BEC, followed by TAD-NP administration. Reprinted with permission from Ref. [16]. Copyright 2024 Springer Nature.

To develop afterglow nanoprobes with high signal intensity, enhanced tissue penetration, and specific biomarker responsiveness, we developed a nanosensing platform incorporating three key components: (1) the enzyme-responsive peptide IEFD, (2) luminescent TAD nanoparticles, and (3) the dark quencher BHQ-3 (Figure 8a). When exposed to granzyme B, the specific proteolytic cleavage of the IEFD linker disrupts the ARET process between TAD-NPs and BHQ-3, leading to significant enhancement of the afterglow emission signal (Figure 8b,c) [16]. Since granzyme B is released by CD8^+^ T cells and natural killer (NK) cells during immune responses, monitoring granzyme B expression levels provides an indirect measure of immune activation following immunotherapy.

**Figure 9:**
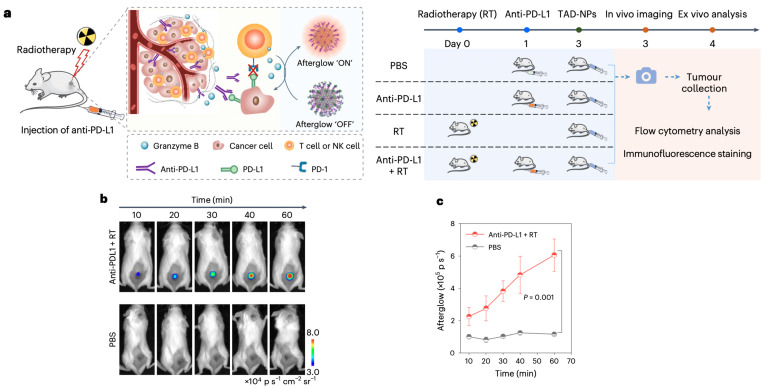
In vivo monitoring of combined radiotherapy and immunotherapy via afterglow luminescence CT-26 tumor models were subjected to four treatment regimens: (i) PBS control, (ii) anti-PD-L1 monotherapy, (iii) radiotherapy (RT) alone, and (iv) combination anti-PD-L1 + RT. After 72 h, all groups received TAD-NP administration for subsequent afterglow visualization. (**a**) Conceptual diagram of TAD-BHQ nanoprobe operation for granzyme B detection post-treatment, including dosing timeline. (**b**) Time-dependent afterglow signals comparing PBS control versus combination therapy groups. (**c**) Quantitative afterglow intensity measurements from tumor regions shown in panel (**b**). Reprinted with permission from Ref. [16]. Copyright 2024 Springer Nature.

Parallel to these investigations, our team assessed immune activation in mice subjected to combined radiotherapy (RT) and PD-L1 blockade by quantitatively monitoring granzyme B expression (Figure 9a). Experimental data revealed markedly enhanced afterglow emission intensities in tumors from the PD-L1 inhibitor plus RT cohort (Figure 9b,c), demonstrating the TAD-BHQ nanosensor’s capability for dynamic assessment and therapeutic evaluation of chemo-radiotherapy and immune checkpoint modulation regimens.

**Figure 10:**
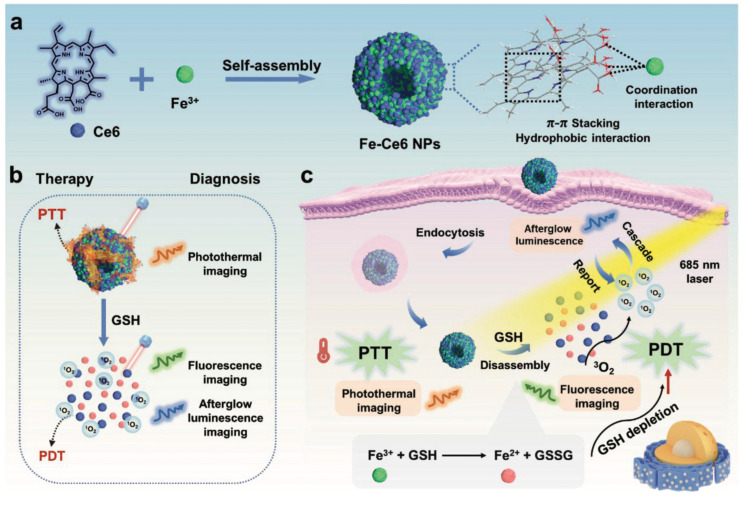
The diagram shows (**a**) the process of preparing Fe-Ce6 NPs. (**b**) The GSH-responsive theranostic application. (**c**) The antitumor effect achieved by combining PTT and PDT. Reprinted with permission from Ref. [50]. Copyright 2023 Wiley-VCH GmbH.

The aforementioned approach, which combines multiple drugs or therapies for synergistic or combination treatment of tumors, is known as combination therapy. Its advantages include reducing drug resistance, inducing apoptosis in cancer cells, and achieving high efficacy, making it a cornerstone of cancer treatment [49]. Pei et al. [50] developed a multifunctional nanotherapy and diagnostic platform for tumor combination treatment by self-assembling Fe-Ce6 nanoparticles (Figure 10a). The designed nanoparticles exhibit two distinct therapeutic mechanisms: they efficiently transform light energy into thermal energy for PTT, while their glutathione (GSH)-sensitive components enable controlled Ce6 release to initiate both photodynamic effects and sustained afterglow emission (Figure 10b,c).

## 6. Conclusions and Outlook

This review offers a critical evaluation of emerging trends and transformative innovations in organic afterglow luminescence research. The article begins by elucidating the mechanisms of afterglow luminescence under various excitation conditions, including light, ultrasound, and X-ray. It then offers detailed examples of several types of organic afterglow materials, such as semiconducting polymers, heterocyclic compounds, and trianthracene derivatives. These materials have attracted significant attention in recent years due to their exceptional biocompatibility and versatile design flexibility. Lastly, the review discusses the cutting-edge applications of organic afterglow nanoparticles in tumor therapy, underscoring their immense potential and promising future in the biomedical field.

Despite the remarkable potential of ultrasound- and X-ray-induced afterglow luminescence systems, current research is confronted with three significant hurdles. (1) The short emission wavelengths of existing nanoparticles impede deep-tissue transmission, restricting high-contrast imaging and precise diagnostic and therapeutic applications; (2) Safety remains a critical concern as the unclear metabolic pathways, unassessed toxicity, and potential immune responses of afterglow materials have stalled clinical trials; (3) Afterglow luminescence is closely associated with oxygen concentration, and hypoxic tumor microenvironments, characterized by low oxygen levels, can quench afterglow signals. In hypoxic conditions, energy transfer and electron transfer mechanisms that rely on oxygen molecules for afterglow luminescence are disrupted, reducing imaging effectiveness.

To address these challenges, future research should focus on three key strategies: (1) Developing systems with extended emission wavelengths to enhance deep-tissue imaging and the capabilities for diagnosis and treatment; (2) Creating safe afterglow materials and establishing comprehensive evaluation systems to promote clinical translation; (3) Incorporating oxygen-supplying molecules via nanocarriers, such as co-delivering nanocarriers loaded with oxygen-releasing compounds alongside afterglow nanoparticles to the tumor site, where the compounds can gradually release oxygen, increase local oxygen concentration, and potentially enhance afterglow luminescence performance in hypoxic tumors.
